# Synthesis of New Amino—β-Cyclodextrin Polymer, Cross-Linked with Pyromellitic Dianhydride and Their Use for the Synthesis of Polymeric Cyclodextrin Based Nanoparticles

**DOI:** 10.3390/polym13081332

**Published:** 2021-04-19

**Authors:** Kinga Kozieł, Jakub Łagiewka, Beata Girek, Agnieszka Folentarska, Tomasz Girek, Wojciech Ciesielski

**Affiliations:** Faculty of Mathematics and Natural Science, Jan Dlugosz University in Czestochowa, Armii Krajowej Avenue, 13/15, 42 201 Czestochowa, Poland; kingakoziel123@gmail.com (K.K.); j.lagiewka17@gmail.com (J.Ł.); b.girek@ajd.czest.pl (B.G.); a.folentraska@ajd.czest.pl (A.F.); t.girek@ajd.czest.pl (T.G.)

**Keywords:** amino-β-cyclodextrin, polymerization, pyromellitic dianhydride, polyampholyte, nanoparticles

## Abstract

New water soluble amino β-cyclodextrin-based polymer was synthesized by reaction between amino cyclodextrin derivatives and pyromellitic anhydride. This experiment presents amino derivatives, which were synthesized by attaching amino groups to β-cyclodextrins (β-CDs) used mono-6-azido-6-deoxy-β-cyclodextrin (β-CD-N_3_) and triphenylphosphine (Ph_3_P) in anhydrous N,N-dimethylformamide (DMF). An amino blocking reaction was conducted. The obtained polymer was purified by ultrafiltration. In addition, an attempt was made to create nanospheres by encapsulating the polymer with chitosan (CT) in an acidic condition. For the first time, nanospheres were obtained in the reaction between an amino β-cyclodextrin polymer and chitosan. Scanning electron microscopy (SEM). ^1^H NMR and ESI-MS methods for confirmation of reaction product and for structural characterization were employed. The differential scanning calorimetry (DSC) studies of polymers were also carried out.

## 1. Introduction

Cyclodextrins (CDs) are cyclic oligomers produced by the enzymatic degradation of starch. CDs are composed of six, seven or eight glucopyranosyl units (named accordingly α-, β- or γ-CDs) linked together by a-1,4-bonds. All CDs are crystalline, homogeneous substances dissolving in water, having the shape of a ring tapering on one side and widening on the other [1]. As a consequence of the chair conformation of glucopyranose mers, all secondary hydroxyl groups at the C2 and C3 are on one of the two edges of the ring and all primary hydroxyl groups at the C6 on the other [2]. Many hydroxyl groups, especially having different properties, allow for the modification of cyclodextrins. They are carried out to expand their applications. Improvements in binding, selectivity or pH allow for the use of modified CDs as chemosensors, artificial enzymes or carriers [3]. The use of cyclodextrins as precursors to form derivatives and polymers is justified because of their biodegradability and total biocompatibility with human tissues [4]. We can also polymerize CDs to give them better stability and lower solubility [5,6,7,8]. The article presents a polymer of a new class of compounds crosslinked with dianhydrides, introducing anionic groups into the polymer network. It is a continuation of our previous work about β-cyclodextrin polymers crosslinked with pyromellitic dianhydride [9]. At the same time, keeping the amino group significantly improves the solubility of the system used. Therefore, a large group of researchers present CD polymers as compounds with potential medical use [10]. The presented polymer material can form hydrogels with many known polycations (chitosan, poly-*L*-lysine) and polyanions (alginic acid, pectin, or hyaluronic acid, etc.) [11,12,13]. β-cyclodextrins and their derivatives are successfully used as material for the formation of microspheres or nanospheres [14,15]. They create biodegradable spheres due to their hydrophobic properties [16,17]. Porous gel spheres with the ability to absorb/release substances [18] contained in them can be used as an additive to drugs to prolong their release [19,20,21]. The same properties allow for the control of fertilization in agriculture as it is broadly understood.

The amino β-cyclodextrin-based polymer contains a large number of anionic and cationic groups. On one hand, the polyelectrolyte properties of the support will facilitate the formation of complexes between the medicinal substances and support, and on the other hand will allow the biomembranes to freely penetrate using the specific properties of polyampholyte. The method of synthesis of a new cyclodextrin polymer having the status of polympholyte was compiled. In this regard, it was synthesized β-CD-NH_2_ by reducing β-CD-N_3_ using triphenylphosphine, followed by hydrolysis to give a product [22]. There was a need to protect the reactive functional group -NH_2_, with a protective group. Used for this a *tert*-butyloxycarbonyl group (BOC) [23,24,25]. The β-CD-NH_2,_ was polymerized using crosslinking reaction [26,27,28]. As a crosslinking agent used pyromellitic dianhydride with NaH. The reaction was carried out under strongly alkaline conditions necessary for deprotonation of glucose hydroxyl groups unit. An ultrafiltration process was used to separate the polymer due to the particle size by 76 mm, 5000 Da membranes selected. Two types of measurement were selected: with molecular weight higher then 5000 Da (PHMW) and with a molecular weight lower then 5000 Da (PLMW). The polymer PHMW was used to create nanoparticles. ESI MS analysis was performed to check the molecular mass of the β-CD-NH_2._ The ^1^H NMR and DSC measurement for the structural characterization of the polymers were conducted. A detailed SEM analysis of several different polymer solutions in a certain concentration range was performed. In previous works [9], a full characterization of similar systems was performed. The presented work is a continuation of the above-mentioned research conducted by our team. Future studies were set up to check the swelling properties and release kinetics of the cross-linked hydrogel membrane; e.g., using the methods described by M.R. El-Aassar et al. [21]. Our new results will be published soon.

## 2. Materials and Methods 

### 2.1. Reagents and Solvents

β-Cyclodextrin (βCD), *N*,*N*-dimethyl formamide (DMF), calcium hydride and sodium hydride were purchased from Sigma-Aldrich, St. Louis, MO, USA. DMF was distilled under vacuum. The dried DMF was stored in a dark bottle over calcium hydride. Sodium hydride (60% in oil) was dried by hexane. Chitosan was purchased from Acros Organics, Pittsburgh, USA, triphenylphosphine (PPh_3_) was purchased from Fluka Analytical, Monte Carlo, USA, pyromellitic dianhydride (PA) was purchased from Alfa Aesar, Ward Hill, Massachusetts, USA. Acetone, *p*-dioxane, acetic acid and hexane were purchased from Chempur, Piekary Slaskie, Poland. Sodium bicarbonate was purchased from Stanlab, Lublin, Poland. Di-tert-butyl dicarbonate (BOC_2_O) was purchased from Merck KGaA, Darmstadt, Germany. Mono-6-azido-6-deoxy-β-cyclodextrin (N_3_-β-CD) was synthesized according to procedure developed by Tang at al. [22]. 

#### 2.1.1. Synthesis of Mono-6-Amino-6-Deoxy-β-Cyclodextrin (AβCD)

Mono-6-azido-6-deoxy-β-cyclodextrin (1.5 g, 1.29 mmol) was dissolved in DMF (2.2 mL). Triphenylphosphine (Ph_3_P) (0.36 g, 1.37 mmol) was added with vigorous stirring for 2 h at room temperature. Later H_2_O (0.26 mL) was added and the solution was heated at 90 °C for 3 h. After this time the reaction mixture was cooled to room temperature. The reaction product was precipitated and washed with acetone (200 mL) [22], and finally dried in a vacuum desiccator at room temperature. Yield: 89%. 

^1^H-NMR. (D_2_O), (δ: ppm): 5.03–4.92 (d, 7H, H-1), 3.95–3.65 (m, 28H, H-3, H-5, H-6), 3.60–3.45 (m, 14H, H-2, H-4), 3.11–3.01 (t, H-6′a), 2.85–2.78 (k, H-6′b). ^1^H-NMR. (DMSO-d6), (δ: ppm): 5.90–5.55 (m, 14H, OH-2, OH-3), 4.95–4.75 (d, 7H, H-1), 4.60–4.35 (d, 6H OH-1), 3.75–3.55 (m, 28H, H-3, H-5, H-6). MS (ESI), (*m*/*z*): Calculated: 1134.3; Found: 1134.27 [M − H]^+^.

#### 2.1.2. Synthesis of Blocking the Amine Group by BOC (BAβCD)

Mono-6-amino-6-deoxy-β-cyclodextrin (1 g, 0.88 mmol) was dissolved in H_2_O (27 mL). Sodium bicarbonate (2 g, 23.81 mmol) was added with vigorous stirring. The solution was cooled to 5 °C and di-tert-butyl dicarbonate (1.5 g, 6.87 mmol) dissolved in solution of *p*-dioxane (5 mL) was added. The mixture was cooled to 2 °C and stirred for 1 h. It was left overnight at room temperature. The product was precipitated with acetone (200 mL) and dried in a vacuum desiccator at room temperature. Yield: 95%. ^1^H-NMR (D_2_O), (δ:ppm): 5.10–4.93 (d, 7H, H-1), 4.13–3.62 (m, 28H, H-3, H-5, H-6), 3.60–3.45 (m, 14H, H-2, H-4), 2.77–2.63 (k, H-6′a), 1,40 (s, 9H, BOC). 

#### 2.1.3. Synthesis of β-CD-NH_2_ Polymer Crosslinked with Pyromellitic Anhydride (PAβCD)

β-CD-NH-BOC (2 g, 1.62 mmol) was dissolved in DMF (20 mL). NaH (0.18 g, 7.5 mmol) was washed with hexane and added in one portion with vigorous stirring. The mixture was stirred for 24 h. After this time, PA (1.74 g, 7.97 mmol) was added in one portion and solution was mixed for another 24 h. The product was precipitated with acetone dried in a vacuum desiccator at room temperature [9]. 

#### 2.1.4. Separation of the Polymer Due to Particle Size

CD polymer sample was dissolved in water and separated by ultrafiltration process at Millipore UF Stirred Cell 76 mm with Ultrafiltration Membrane, Regenerated Cellulose PLCC 5000 Da. The pressure of nitrogen gas was 2.2 bar. The separated fractions were recovered and weighed.

#### 2.1.5. The Preparation of Nanospheres

The 0.1% solution of chitosan (25 mL) in 1.75% acetic acid and a 0.1% aqueous solution of the resulting polymer (25 mL) was prepared. 5 mL chitosan solution were poured into 5 bottles and 0.25, 1, 2, 2.5 and 3 mL polymer solution were added in succession under magnetic stirring at room temperature [16]. 

#### 2.1.6. NMR Measurement

^1^H-NMR (600 MHz) spectra were recorded on a Avance II Bruker Ultrashield Plus spectrometer in a 5 mm sample tube, using D_2_O and DMSO as the solvents. All spectra were obtained at ambient temperature. 

#### 2.1.7. ESI-MS Experiment

The mass spectra (ESI-MS) were recorded on a Thermo Finnigan LCQ Fleet (Thermo Fisher Scientific Inc., San Jose, CA, USA) mass spectrometer.

#### 2.1.8. SEM Measurement

Scanning electron microscopy (SEM) of polymer were evaluated on Vega 3, Tescan. All samples were subjected to 4 kV beam energy. Samples of nanoparticles were analyzed by a Nova Nano SEM 200 microscope of up to 2 nm resolution and 70–500 000 × magnification equipped with a field FEG Schotky emitter (FEJ Europe Company, Hillsboro, OR, USA). All samples were subjected to 5 kV beam energy. 

#### 2.1.9. DSC Measurement

Differential scanning calorimetry (DSC) were recorded on a NETZSCH STA-409 simultaneous thermal analyzer (Selb, Germany), calibrated with standard aluminium of 99.99% purity. A sample of the tested material in corundum crucibles with non-hermetic lids was placed in a measuring chamber and heated in the temperature range 20–500 °C with the 5 °C min/L temperature rate. The measurements were duplicated. Recorded thermograms were analyzed with the NETZSCH-TAANALYSIS program. 

## 3. Results and Discussion

### 3.1. Synthesis of PAβCD

**Figure 1:**
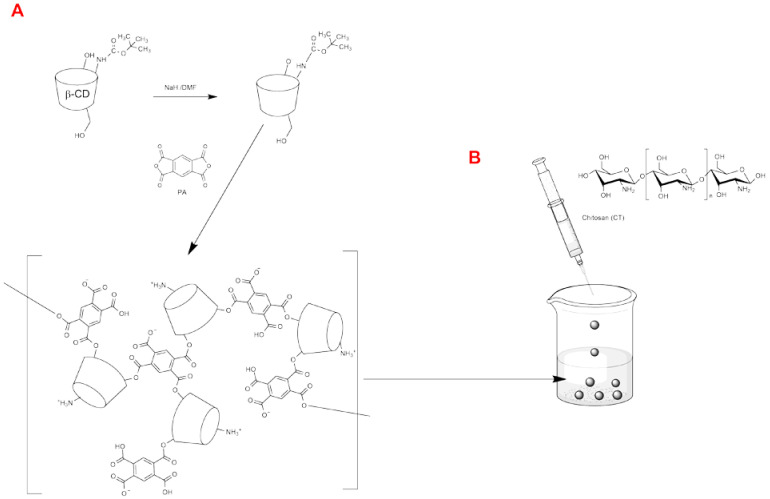
The scheme of polymer synthesis (**A**) and formation of spheres (**B**).

In these studies pyromellitic dianhydride was used as a linker to synthesize polymer with a significant amount of anionic and cationic groups. Crosslinking reaction took place by deprotonating the hydroxyl groups at the C2-position with NaH attaching PA at this position [29]. Figure 1A illustrates this process. The molar ratio of the reactants was 1:4:4. Deprotonation and crosslinking took place in dry DMF. The solution turned to a gel upon addition of PA. 

### 3.2. Separation of the Polymer Due to Particle Size

Samples obtained in this reaction were initially separated by ultrafiltration at Milipore UF Stirres Cell equipped with Ultrafiltration Membrane with the cut-off size of 5000 Da. Table 1 represents the results of the ultrafiltration experiment.

The experiment showed a quantitative advantage of PLMW over PHMW. We obtain PHMW in 25%, and PLMW in 51% yield, respectively. In the general sample, the mass of PLMW is twice as large as the mass of PHMW.

### 3.3. The Preparation of Nanospheres

The spheres were made using ionic gelation between the newly formed polymer and chitosan. The chitosan solution was dripped into the polymer solution with a syringe and needle. The formed drop hardened by falling into the solution. Nanospheres were created from the same reaction using an atomizer. The spheres were made with using a PHMW. Figure 1B illustrates this process.

### 3.4. Spectroscopic Characterization 

**Figure 2:**
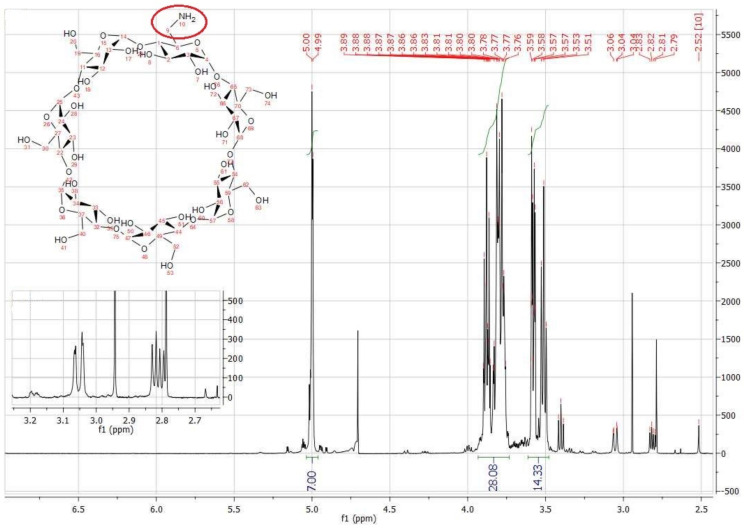
The ^1^H NMR spectra of AβCD.

The ^1^H NMR spectra of AβCD is shown in Figure 2. D_2_O was used as the solvent. The most important part of the spectrum presented are the two signals coming from the H9 protons resonance, the carbon-nitrogen bond with the amino group. They are respectively: 3.05 ppm (dd) and 2.81 ppm (dd). 

### 3.5. Spectrometric Characteristics

**Figure 3:**
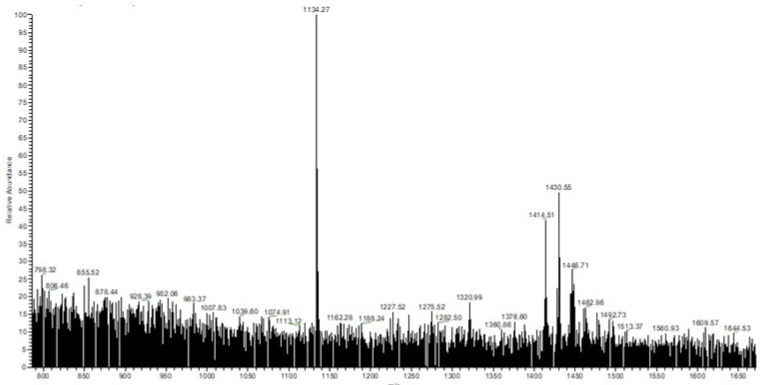
Fragment of the mass spectra (ESI-MS) of AβCD.

The mass spectra (ESI-MS) of AβCD is shown in Figure 3. On the fragment of the spectrum, here is a visible parent peak with the value *m*/*z* = 1134.27 (M + H^+^), corresponding to the literature values for AβCD: 1134.30.

### 3.6. SEM Characterization 

The structure of polymer was analyzed with the Scanning Electron Microscopy, samples βCD, PAβCD, PLMW and PHMW were analyzed, respectively. Nanospheres made of PHMW were also analyzed. 

**Figure 4:**
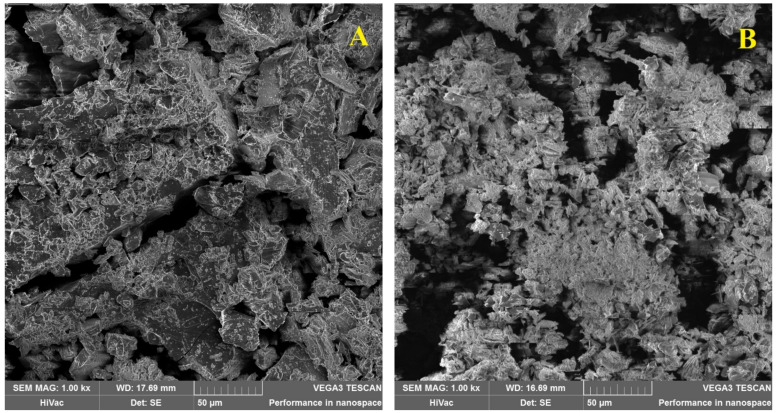
SEM imagines of (**A**) the βCD (1000× zoom) (**B**) the PaβCD (1000× zoom).

**Figure 5:**
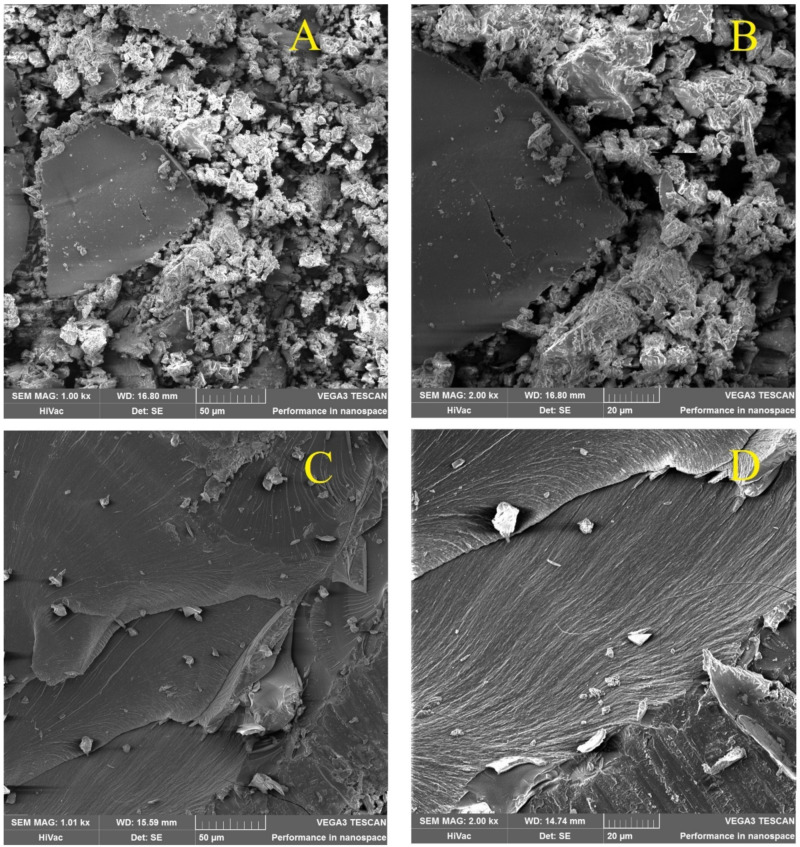
Crystal structure of (**A**) PLMW (1000× zoom), (**B**) PLMW (2000× zoom), (**C**) PHMW (1000× zoom), (**D**) PHMW (1000× zoom).

Figure 4 shows morphologies of βCD monomer (A). It has a non-smooth surface with cracks, which can be attributed to the crystallinity of cyclodextrin. On the other hand, the right photo presents the PAβCD. (B). The polymer particles are packed in a network with a large number of micropores. The polymer contains clusters of separate particles and has larger holes, but the surface of a single particle is tightly packed with small holes. SEM analysis of the polymer after ultrafiltration was also performed. At Figure 5A,B is shown the crystal structure of the PLMW. The structure resembles PAβCD. The same figure also shows large crystals from the high molecular product, most likely because of incomplete ultrafiltration. Figure 5 C,D shows the crystal structure of the PHMW which has a smooth surface, and the particles are packed much tighter. The pores are less visible.

**Figure 6:**
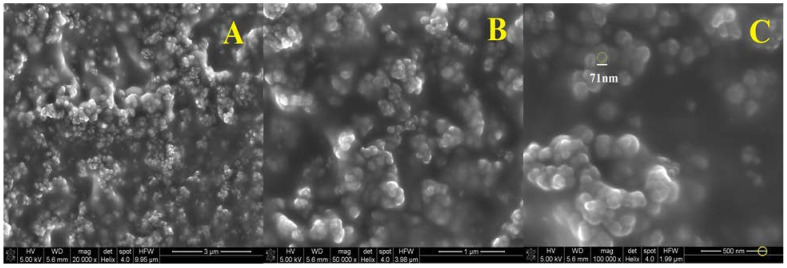
SEM imagines of nanospheres suspension 1 enlarged 20,000 times (**A**), 50,000 times (**B**) and 100,000 times (**C**).

**Figure 7:**
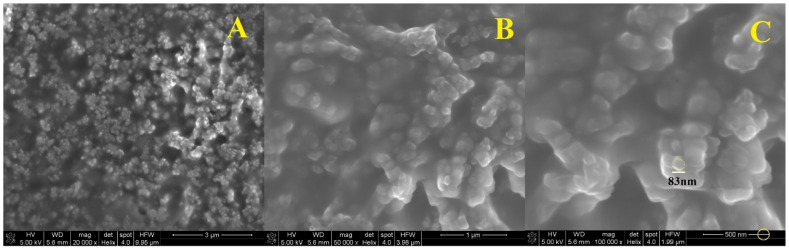
SEM imagines of nanospheres suspension 2 enlarged 20,000 times (**A**), 50,000 times (**B**) and 100,000 times (**C**).

**Figure 8:**
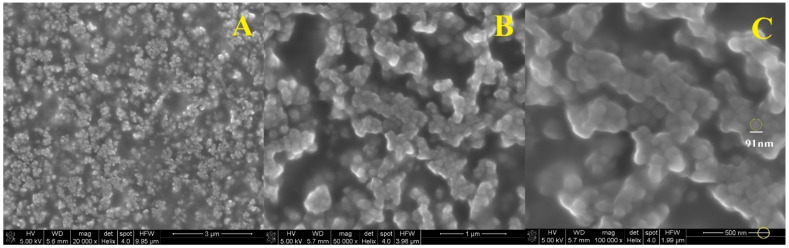
SEM imagines of nanospheres suspension 3 enlarged 20,000 times (**A**), 50,000 times (**B**) and 100,000 times (**C**).

SEM analysis was also performed for three solutions containing nanospheres. Samples of the finished suspension containing 0.25 (1), 1 (2) and 2 (3) mL solution of (0.1%) polymer in acidic solution (0.1%) of chitosan were used for the test. The solution concentrations were 5%, 25% and 40%, respectively. As shown in Figure 6, Figure 7 and Figure 8, the SEM characterization confirmed the formation of nanospheres. Pictures with magnification of 20,000 (A), 50,000 (B) and 100,000 times (C) were taken for each solution. 

It can be seen that the polymer in combination with chitosan gives spherical particles with a relatively homogeneous surface. Examination of the samples showed that the spheres in the solution are in the form of larger aggregates, and it was not possible to obtain a suspension of separate spheres. In Figure 6 is the SEM photo of suspension 1, where the diameter of one nanosphere was about 71 nm (Figure 6C). The morphology of suspension 2 showed that the particle size was about 83 nm (Figure 7C). The diameter of the sphere from Figure 8C was about 91 nm (suspension 3). The size of the nanospheres depends on the concentration of the polymer in chitosan. The higher concentration gives the larger particle diameter. 

### 3.7. Differential Scanning Calorimetry Analysis 

**Figure 9:**
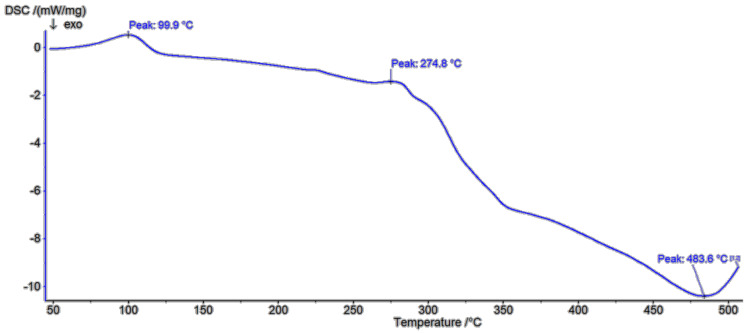
DSC spectra of β-cyclodextrin.

**Figure 10:**
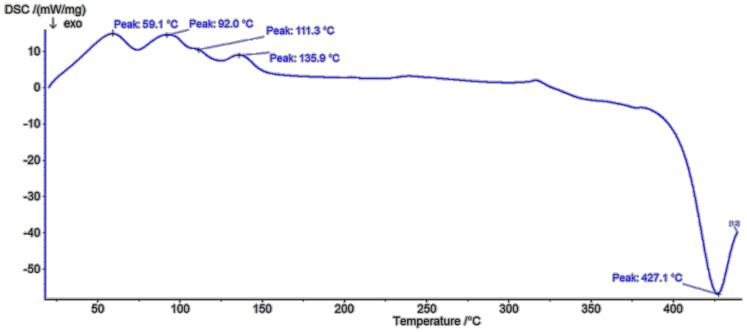
DSC spectra of PLMW.

**Figure 11:**
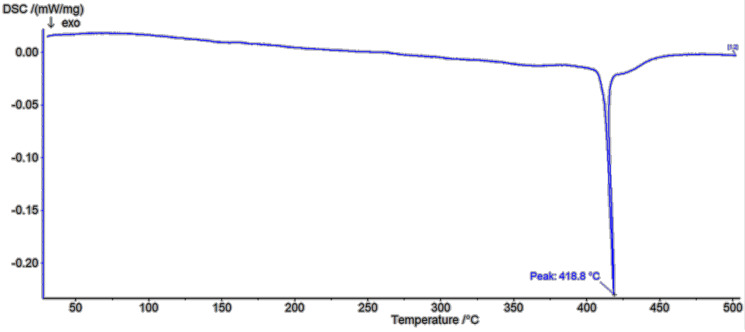
DSC spectra of PHMW DSC spectra of high molecular weight polymer.

In the β-CD DSC diagram (Figure 9) two melting peaks at 99.9 °C and 274.8 °C were found to be related to oxidation or elimination of water [30]. In the first one, the weight loss was 8.64%, with the next at 50.18%. The shape of corresponding parts of DSC diagram of low molecular weight polymer (Figure 10) confirms a different type of water coordination (compared to pure β-CD), as well as its three-stage removal. This is indicated by the presence of small peaks at about 59.1 °C, 92 °C and 135.9 °C. In the study of the high molecular weight polymer (Figure 11) a glass transition point at about 418.8 °C was demonstrated. In all cases, the exothermic effects had a different maximum. As the molecular weight increased, the maximum of the exothermic effects decreased. 

## 4. Conclusions

New, water soluble, polyampholyte CD polymer was prepared by crosslinking amino-β-cyclodextrin with pyromellitic dianhydride. The ^1^H NMR and ESI-MS studies confirmed the products of indirect reactions. The separation of the polymer due to particle size by ultrafiltration process was employed. The division of the polymer with respect to the molecular weight showed an unequal mass ratio between the high and low molecular weight product. The analysis of the formed polymer showed a change in surface area relative to pure β-cyclodextrin. For the first time, nanospheres were obtained in the reaction between an amino β-cyclodextrin polymer and chitosan. The SEM imagines of nanospheres confirmed the ionic gelation of the polymer with chitosan. Obtained nanospheres having a diameter of less then 90 nm SEM analysis showed the formation of nanoparticles, the diameter of which varies with the concentration of the polymer. Nanospheres can be precursors for the encapsulation of substances used in pharmacology and agriculture.

## Figures and Tables

**Table 1:** Ultrafiltration data for samples prepared of AβCD, NaH and pyromellitic dianhydride.

Cut-Off	Weight [g]	% Of Whole Sample
Whole sample	2.00	100
PLMW	1.02	51.00
PHMW	0.50	25.00

## Data Availability

Data is contained within the article.
